# Surviving winter: Food, but not habitat structure, prevents crashes in cyclic vole populations

**DOI:** 10.1002/ece3.2635

**Published:** 2016-12-14

**Authors:** Kaja Johnsen, Rudy Boonstra, Stan Boutin, Olivier Devineau, Charles J. Krebs, Harry P. Andreassen

**Affiliations:** ^1^ Faculty of Applied Ecology and Agricultural ScienceHedmark University of Applied SciencesKoppangNorway; ^2^ Department of Biological SciencesUniversity of Toronto ScarboroughTorontoONCanada; ^3^ Department of Biological SciencesUniversity of AlbertaEdmontonABCanada; ^4^ Department of ZoologyUniversity of British ColumbiaVancouverBCCanada

**Keywords:** climate, feeding experiment, habitat quality, population dynamics, rodents

## Abstract

Vole population cycles are a major force driving boreal ecosystem dynamics in northwestern Eurasia. However, our understanding of the impact of winter on these cycles is increasingly uncertain, especially because climate change is affecting snow predictability, quality, and abundance. We examined the role of winter weather and snow conditions, the lack of suitable habitat structure during freeze‐thaw periods, and the lack of sufficient food as potential causes for winter population crashes. We live‐trapped bank voles *Myodes glareolus* on 26 plots (0.36 ha each) at two different elevations (representing different winter conditions) in southeast Norway in the winters 2013/2014 and 2014/2015. We carried out two manipulations: supplementing six plots with food to eliminate food limitation and six plots with straw to improve habitat structure and limit the effect of icing in the subnivean space. In the first winter, all bank voles survived well on all plots, whereas in the second winter voles on almost all plots went extinct except for those receiving supplemental food. Survival was highest on the feeding treatment in both winters, whereas improving habitat structure had no effect. We conclude that food limitation was a key factor in causing winter population crashes.

## 1 Introduction

Small mammal populations in the northern hemisphere often show cyclic dynamics in their abundance (Hanski, Hansson, & Henttonen, 1991; Kendall, Prendergast, & Bjornstad, 1998; Steen, Yoccoz, & Ims, 1990). Vole populations in Fennoscandia show conspicuous 3–4 year cycles (Boonstra et al., 2016). Many hypotheses have been proposed to explain these cycles (for a recent review, see Andreassen, Glorvigen, Rémy, & Ims, 2013), but high predation, low food availability, and possibly intrinsic factors (e.g., infanticide) predominate.

Population cycles are characterized by a low, increase, peak, and crash phase, each lasting approximately 1 year (Andreassen et al., 2013). The crash phase may occur during the breeding season (Andreassen et al., 2013; Stenseth & Ims, 1993), but more often is observed during winter (Hansson & Henttonen, 1985; Krebs & Myers, 1974). In Fennoscandia, voles spend about 4–6 months per year living under the snow. Stable subnivean conditions are crucial for good survival as warm and wet winters can lead to frequent melting‐freezing events that can limit vole access to food by encasing it in ice. Icing can also affect vole movements by splitting up the subnivean space into accessible and inaccessible parts (Aars & Ims, 2002; Hörnfeldt, 2004; Kausrud et al., 2008; Korslund & Steen, 2006). In addition to the conditions in the subnivean space, snow also insulates and protects voles against predators (Lindström & Hörnfeldt, 1994). Winter climate and snow or icing conditions may therefore directly or indirectly affect vole population dynamics (see e.g., Huitu, Koivula, Korpimäki, Klemola, & Norrdahl, 2003). The lack of peak years in the last 20–30 years and hence lack of cycles in many vole populations in Fennoscandia have been attributed to consistently poor overwinter survival owing to adverse winter conditions (Cornulier et al., 2013; Hörnfeldt, 2004; Ims, Henden, & Killengreen, 2008; Kausrud et al., 2008).

Most of our knowledge of vole population cycles in Fennoscandia is based on longitudinal data in which animals were trapped twice a year, in spring and fall (Ehrich, Yoccoz, & Ims, 2009; Hansen, Stenseth, & Henttonen, 1999; Hörnfeldt, 2004). We lack detailed studies following vole populations during winter that can pinpoint when poor survival occurs and link this to an extrinsic cause. To understand the cause of poor overwinter survival in Fennoscandia, we intensively live‐trapped bank voles *Myodes glareolus* throughout two winters. These populations were located at two different elevations as a means of assessing the impact of different winter climate conditions. In addition, we performed two experimental manipulations to increase winter survival. First, we improved habitat structure by adding straw (for insulation and prevention of ground‐level icing). Second, we added food to assess whether food was limiting overwinter. For two consecutive winters, one of which experienced a severe vole population crash, we followed bank voles on 26 different trapping plots.

We tested three hypotheses. (1) The Winter Stability Hypothesis: Winter weather and snow conditions have a direct effect on vole survival. This would be observed as an elevation effect on survival rates with a lower survival at low elevation where freeze‐thaw events are more frequent. (2) The Subnivean Habitat Structure Hypothesis: This is an indirect consequence of winter weather and snow conditions mediated through lack of accessible habitat structure. If this is correct, supplemented straw would have a positive effect on survival. (3) The Food Limitation Hypothesis: Lack of accessible food is an indirect consequence of winter weather and snow conditions. If this is correct, supplemented food would have a positive effect on survival.

## 2 Material and Methods

### 2.1 Study animal

The bank vole is a small microtine rodent distributed across Europe from mature forests to reforestation areas and meadows (Mitchell‐Jones et al., 1999; Myllymäki, 1977). In Fennoscandia, reproduction mainly occurs during the summer season from late April to October (Koivula, Koskela, Mappes, & Oksanen, 2003). Females are territorial whereas males are not, with home ranges being large and overlapping extensively (Bujalska, 1973; Mazurkiewicz, 1971). Female territoriality is assumed to be a response to the spatial distribution, abundance, and renewal of food resources (Boonstra & Rodd, 1983; Ostfeld, 1990). The winter diet of bank voles in Fennoscandia is dominated by dwarf shrubs *Vaccinium* spp., but do also includes fungi, chordate lichens, and some berries and seeds (Hansson & Larsson, 1978). Hansson and Larsson (1978) found evidence of decreasing amounts of seed and berries in their diet during the crash phase. Voles in the subnivean space are vulnerable to predation by specialist predators such as the stoat *Mustela erminea* and the least weasel *M. nivalis* (Korpimäki, Norrdahl, & Rinta‐Jaskari, 1991). If subnivean ice drives the voles above the snow or if snow cover is absent, voles will also be vulnerable for predation by the generalist red fox *Vulpes vulpes* (Lindström & Hörnfeldt, 1994) or the specialist avian predators such as the Tengmalm's owl *Agolius funereus* (Korpimäki, 1994).

### 2.2 Study area

**Figure 1:**
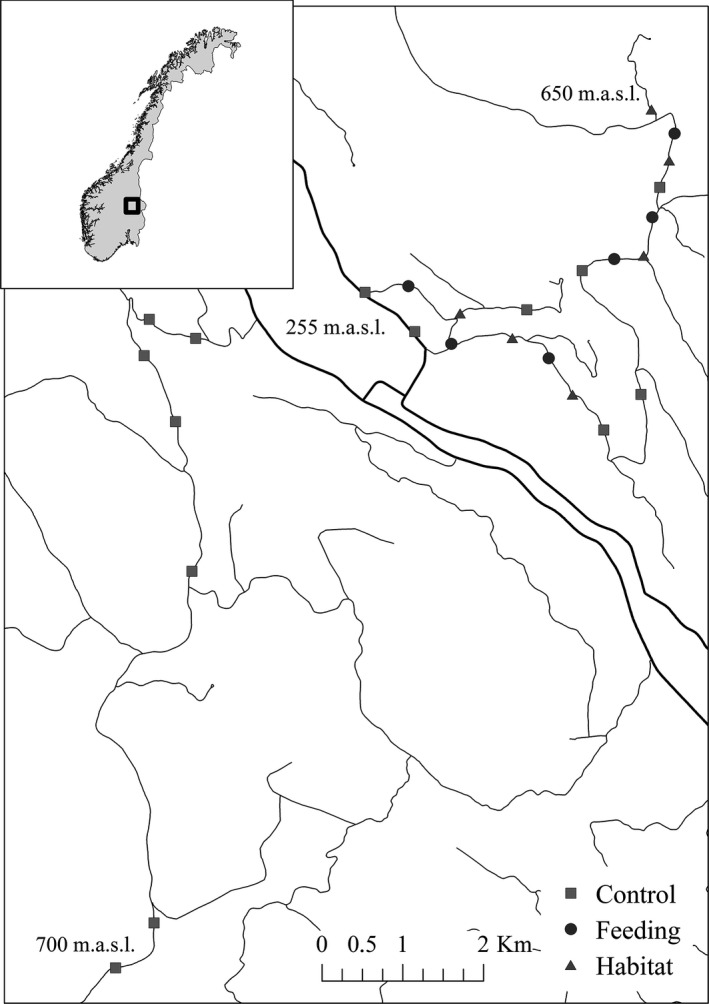
Map of the study area in SE Norway where winter survival of bank voles was studied over two winters (2013/2014 and 2014/2015). The detailed map shows trapping plot design of Control (*n* = 14), supplemental Feeding (*n* = 6), and Habitat structure (*n* = 6) along roads. The black thick lines surrounding the label “255 m.a.s.l.” indicate main roads in the valley bottom

We carried out the experiment in the boreal forests of Stor‐Elvdal municipality in southeast Norway (61°N, 11°E) (Figure 1) in the winters 2013/2014 and 2014/2015. These forests are dominated by Norway spruce *Picea abies* and Scots pine *Pinus sylvestris*, with bilberry *Vaccinium myrtillus* in the understory shrub layer, and mosses (*e.g., Pleurozium schreberi*) in the ground layer. The region has experienced dampened cycles and the absence of peak years of voles and lemmings since the mid 1980s (Hörnfeldt, 2004). In 2007, the peaks returned and have been regular since then (summer peaks in 2007, 2010/2011, and 2013/2014—unpublished data from Hedmark University of Applied Sciences and the data we present here).

Annual precipitation in the study area averaged 571 mm and the annual temperature (1971–2015) averaged 2.9°C at low elevation and 0.6°C at high elevation (data obtained from the Norwegian Meteorological Institute, Evenstad weather station at 257 m.a.s.l. and Drevsjø weather station at 672 m.a.s.l.). January and July temperature averaged –9.0°C and 15.0°C, respectively, at low elevation and –9.9°C and 12.6°C, respectively, at high elevation. The minimum temperature observed since 1971 was −37°C at low elevation and −47°C at high elevation. On average snow covers the ground from November/December to April at low elevation and October to May at high elevation. Snow depth may be up to 1.35 m at both sites.

### 2.3 Trapping procedure

**Figure 2:**
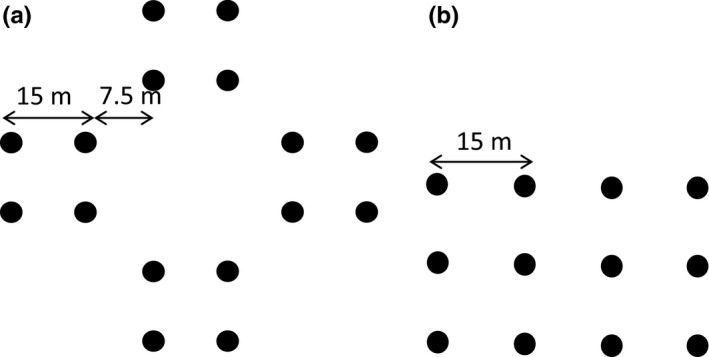
Trapping plot design. The left panel shows the main, cross‐shaped design with 16 traps, and the right panel the alternative design used when the main design did not encompass any suitable vole habitat, with 12 traps

Voles were caught on 60 m × 60 m plots consisting of 16 Ugglan multiple capture live traps (Granab, Sweden) arranged in a cross‐pattern (spacing between traps 15 m) (Figure 2a), except for four plots where we adjusted the layout in order to encompass suitable habitat (Figure 2b). Plots were located in typical bank vole habitat, preferably in mature forest with areas dominated by bilberry in the understory shrub layer (Gorini et al., 2011; Myllymäki, 1977), and near a forest road. We placed plots in all suitable forest habitat fragments along the road, but with a minimum of 500 m between plots.

To increase capture probabilities, traps were located close to a runway or a hole with potential vole activity within a 3 m radius from the predefined layout of traps (i.e., cross‐pattern). Traps were left at the capture site permanently so that voles could habituate to them and use them as part of their runway system when traps were not active. Each trap was covered with a 30 × 30 × 40 cm floorless plywood box to prevent the traps from being covered with snow. The boxes were removed in the spring when the snow melted around them. Trap locations were marked with a stick and a ribbon in the closest tree, and they remained fixed throughout the study.

During a live‐trapping session, traps were set in the evening of day 1, checked the next morning, and evening each day for 3 days (six secondary trap occasions per session). Traps were supplemented with sawdust for warmth and baited with oats and carrots. On the Control plots, trapping occurred once a month from October to May 2013/2014 and 2014/2015. On the experimental plots, trapping occurred only once in the autumn and once in the spring. In January–April 2014, some trap‐days were lost owing to either extreme cold (below −20°C) or to heavy snow concealing the traps. Captured voles were individually marked with pit‐tags (1.25 × 7 mm ID‐100VB Nano Transponder), sexed, weighed to the nearest gram, and checked for reproductive status (mature if open vagina or scrotal testicles). We used a basic LID‐560 Pocket Reader (Trovan) to read the tags.

### 2.4 Experimental design

Our experimental design assessed the impact of elevation, food, and habitat structure on vole survival and dynamics. The manipulations were duplicated at two elevations (low elevation: 280–320 m.a.s.l., high elevation: 550–700 m.a.s.l.) to permit comparison of vole population performance under conditions that were expected to be more stable and less subject to temperature fluctuations and icing (high elevation), and less stable (low elevation).

The Feeding experiment was designed to prevent winter food limitation and the Habitat experiment was designed to create an ice‐free subnivean habitat structure. On the Feeding plots, we provided a mixture of 80 % oats and 20 % sunflower seeds ad libitum inside the trap boxes. We regularly checked the food during the winter and added some if necessary. We use a total of ca. 250 kg of seeds per winter. For the Habitat plots, we spread straw 20 cm thick over about 4 square meters centered on each of the trap boxes. Hence, each of the 16 trap stations on a plot received this amount of straw.

At each elevation, trapping plots for Control, Feeding, and Habitat were randomized along the forest roads. We had eight Control plots at low elevation and six at high elevation, and three Feeding plots and three Habitat plots at each elevation, making a total of 26 plots. Each year, the Feeding and Habitat manipulations were initiated in November and lasted until May, when the snow had melted enough to expose bare ground at both elevations.

### 2.5 Winter conditions

Temperature loggers (HOBO U23 Pro V2) were used to record subnivean temperature every 6 hr in 10 plots: five at high elevation and five at low elevation. Snow depth (measured to the nearest cm), the presence of snow crust layers, and the presence of icing on the ground were determined once every trapping session each time the Control plots were trapped. Snow crust layer was assessed as the presence/absence of one or several snow crust layers. Icing on the ground was assessed as the presence/absence of ice on the ground.

### 2.6 Data analysis

#### 2.6.1 Comparison of winter conditions

Using plot as a grouping factor, we compared each winter climatic variable between years and elevations with a generalized linear mixed model (GLMM), using either a Gaussian (daily mean temperature, snow depth, average subnivean temperature) or a binomial error distribution (presence/absence of snow crust and icing). All analyses were performed in R (R Core Team 2016).

#### 2.6.2 Capture–recapture data analysis

We analyzed the capture–recapture data using the robust design approach (Kendall, 1999; Pollock, 1982). It assumes that the population is open between primary trapping sessions (i.e., from one month to the next), but closed within trapping sessions (i.e., the secondary occasions from one trap check to the next during a given trapping session). This allows the model to provide estimates for (monthly) true survival S and abundance N, as well as for the capture/recapture (denoted p and c) and emigration/return (γ” and γ') probabilities.

Because our study occurred during the decline and low phase of the vole population cycle, too few animals were captured on each plot to permit including variation among plots in our models. We simply give an indication of this variation by listing the minimum number of voles known to be alive in the Appendix. Nonetheless, our main interest was to compare the Treatment factors (Control, Feeding, and Habitat). Pooling animals across plots, that is, ignoring among‐plot variation, allowed us to model the differences in survival and abundance among the three treatments and two elevations. We scaled the abundance estimates before the comparison to account for the fact that the number of plots varied between Control and manipulated plots, that is, we divided the abundance estimate by the number of plots for each treatment and elevation.

In addition to treatment and elevation, we were interested in how vole abundance changed throughout the winter. However, monthly trapping was carried out only at the Control plots. We therefore analyzed the Control data alone to obtain monthly estimates of abundance. We carried out a second analysis in which we discarded all but the first and last trapping sessions (i.e., December/November and May) of the Control data to permit comparison to the Treatment data. With this alternative parameterization, we estimated abundance in autumn and in spring, as well as survival over 6‐month periods (i.e., from November/December to May).

We fitted all models using the program MARK (White & Burnham, 1999) via the RMark interface (Laake, 2013) in R (R Core Team 2016). We performed model selection based on the Akaike's information criterion, corrected for small samples (AICc). For the monthly abundance on the Control plots, we modeled survival as an effect of elevation, time (monthly), and their interaction. We considered the capture and recapture probabilities dependent on time or on elevation. For all models, we set emigration and return to be equal and random. For the 6‐month survival and abundance for Treatment and Control, we modeled survival as an effect of time (6 months), Elevation and Treatment, as well as their three‐way interaction and all possible two‐way interactions. We set capture and recapture probabilities to be treatment dependent across all models, whereas emigration and return were set to be equal and random.

Because of the small sample sizes (especially for the 2014/2015 winter), we encountered some convergence problems and parameters approached their lowest level. We therefore re‐fitted our best AIC model with the Markov Chain Monte Carlo (MCMC) estimation procedure available in program MARK (with 30,000 iterations on three chains). This helped us obtain parameter estimates (and their distribution) together with their associated 95 % highest posterior density intervals which we used to compare estimates between autumn/spring and between treatments. For model selection, we chose to present results from the model with highest AIC weight. If there were several best models, that is, low ΔAIC and similar AIC weights, we selected the simplest of the best models (i.e., the model with the fewest parameters).

## 3 Results

### 3.1 Winter conditions

**Figure 3:**
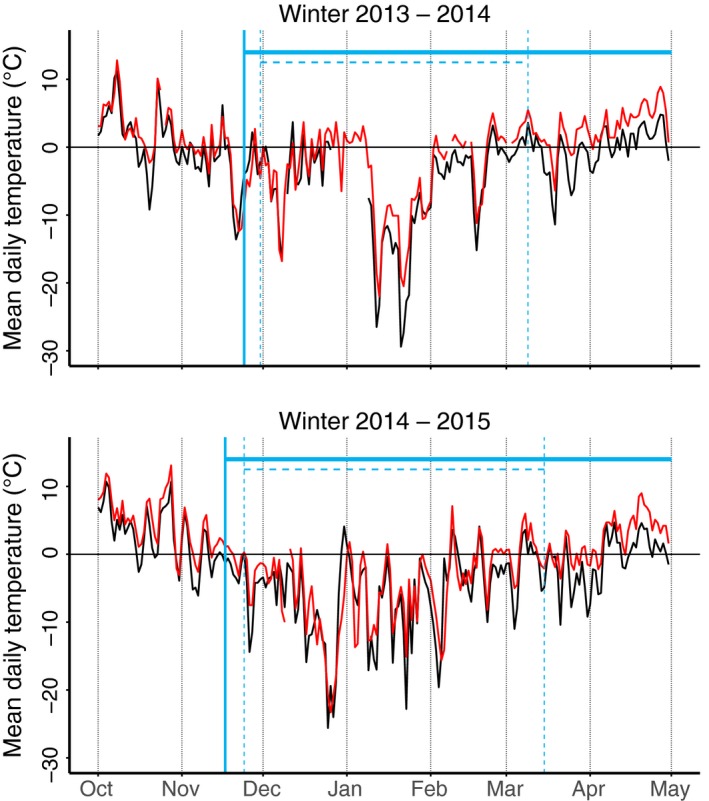
Daily temperature (°C) variation at high (black lines) and low (red lines) elevation during winters 2013/2014 and 2014/2015. The periods with the presence of a continuous snow cover are shown with blue vertical lines in high (continuous lines) and low (dashed lines) plots

The temperature and snow cover profile was similar during the two winters (Figure 3). Snow covered the ground completely in mid‐November in both years and lasted until mid‐March on the low plots and until May on the high plots. A 10‐day mild period from the end of December 2013 to early January 2014, resulted in exposed ground reappearing and thus in fragmented snow cover on the low elevation plots.

On the trapping plots, the air temperature averaged 2.1°C (SE = 0.7) higher on low plots than on high plots (F_1,30_ = 89.77, *p* < .001). Snow depth measurements in the sessions was 18.1 cm (SE = 4.9) deeper in the winter of 2013/2014 than in the winter of 2014/2015 ($\chi_{1,25}^{2}$ = 14.22, *p* < .001), and 22.4 cm (SE = 5.0) deeper on the high than on the low elevation plots ($\chi_{1,25}^{2}$ = 8.49, *p* = .004). The proportion of plots that had snow crusts during a trapping session was greater in 2014/2015 than in 2013/2014 ($\chi_{1,26}^{2}$ = 4.81, *p* = .028). However, there were no differences in mean subnivean temperature or proportion of plots with the presence of icing on the ground (all *p* > .2) (Table 1).

**Table 1:** Mean (95% CI) weather conditions in southeastern Norway during the winter period with snow cover from December 1 until April 30 at high (550–700 m.a.s.l.) and low (280–320 m.a.s.l.) elevation during the winters of 2013/2014 and 2014/2015

	Winter 2013/2014		Winter 2014/2015	
	High	Low	High	Low
Mean temperature T (°C)	−3.6 (−5.1, 2.1)	−1.1 (−2.4, 0.2)	−4.3 (−5.5, −3.2)	−2.6 (−3.9, 1.3)
Mean subnivean temperature (°C)	−0.9 (−1.7, −0.1)	−0.2 (−0.9, 0.5)	−0.6 (−1.4, 0.1)	−0.4 (−1.1, 0.3)
Mean snow depth (cm)	78.6 (65.9, 91.3)	52.5 (41.6, 63.5)	56.0 (43.9, 68.1)	37.6 (27.1, 48.1)
Percentage of plot‐sessions with snow crust	81 (61, 92)	69 (52, 82)	83 (66, 93)	92 (79, 98)
Percentage of plot‐sessions with icing	8 (2, 26)	17 (8, 33)	13 (5, 31)	15 (7, 30)

### 3.2 Abundance

**Figure 4:**
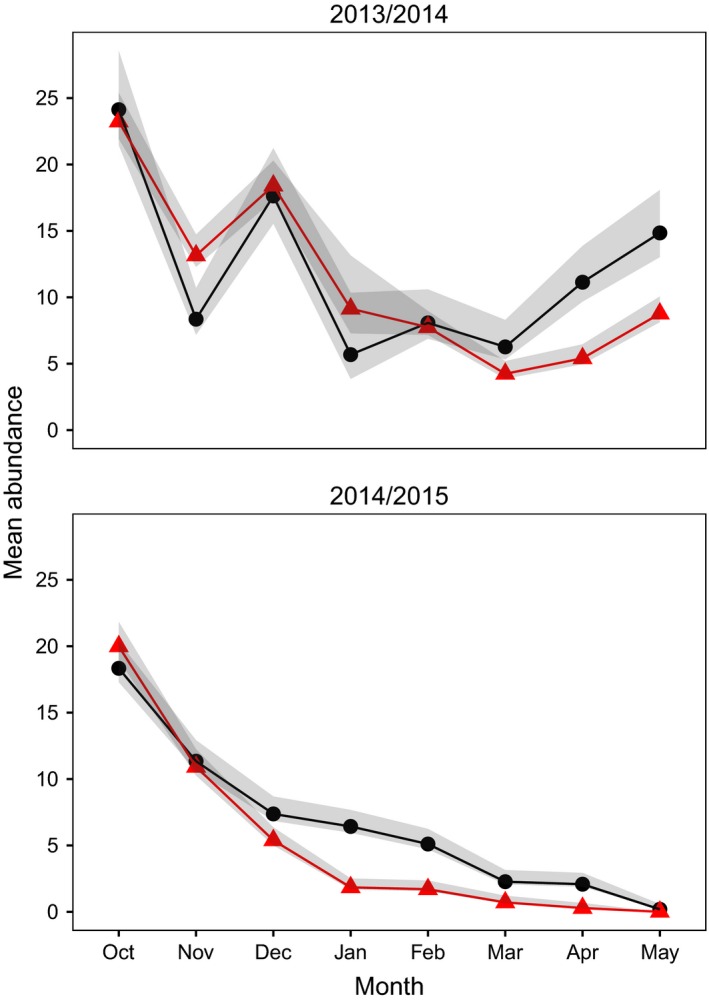
Mean monthly abundance of bank voles with 95 % CI per plot (3600 m^2^) for Control populations only during the winters 2013/2014 and 2014/2015. The high elevation populations are presented with black circles and lines and the low elevation populations with red triangles and lines. Estimates for the top and bottom panel were, respectively, obtained from the model highlighted in the first and second part of Table 2

A total of 1,151 individual voles were captured 7,479 times during the two winters of study. We observed no reproduction, that is, no weanlings were captured, nor were any females lactating or pregnant. The abundance on the Control populations differed over the two winters (Figure 4). Control populations started with a higher mean autumn abundance in 2013/2014 (24 individuals per plot, i.e., per 3,600 m^2^) than in 2014/2015 (19 individuals). After the 2013/2014 winter, Control populations had a mean of 12 individuals in spring. In contrast, after the 2014/2015 winter, the Control populations declined continuously to a mean abundance of 0.1 individuals by spring. The most parsimonious model selected to explain the change in abundance over the winter on Control populations showed that survival was higher on high than on low populations for both years, whereas capture and recapture probabilities were higher on low than on high populations in 2013/2014 (Tables 2 and 3).

**Table 2:** Model selection for the modeling of monthly winter abundance of bank voles on Control populations. We used the robust design model with Huggins parameterization. The two winters, 2013/2014 and 2014/2015, were analyzed separately. Only the five best AIC models are shown for each analysis, and the selected model is highlighted. For 2013/2014, we selected the model with highest weight, whereas in 2014/2015, we selected the simplest model of the two highest rank models. The model structure for GammaPrime (γ'), GammaDoublePrime (γ”) are not shown, because they were the same across all models (γ' = γ”(time), that is, random emigration), p: probability of initial capture during a trapping session, c: probability of recapture during a trapping session, conditional on initial capture. Elevation is the elevation level (low, high); Time corresponds to the monthly primary trapping sessions

Model	npar	AICc	ΔAICc	AICc weight	Deviance
Winter 2013/2014					
**S(Elevation)p(Elevation)c(Elevation)**	**13**	**9353.57**	**0.00**	**0.72**	**11155.79**
S(Elevation)p(Elevation)c(1)	12	9355.54	1.97	0.27	11159.78
S(Elevation)p(1)c(Elevation)	12	9364.53	10.96	0.00	11168.77
S(Elevation)p(1)c(1)	11	9366.50	12.93	0.00	11172.76
S(Time)p(Elevation)c(Elevation)	18	9369.75	16.18	0.00	11161.83
Winter 2014/2015					
S(Elevation)p(Elevation)c(1)	12	5491.17	0.00	0.34	6474.79
**S(Elevation)p(1)c(1)**	**11**	**5491.22**	**0.05**	**0.33**	**6476.88**
S(Elevation)p(Elevation)c(Elevation)	13	5493.16	1.99	0.12	6474.75
S(Elevation)p(1)c(Elevation)	12	5493.21	2.04	0.12	6476.83
S(Time)p(1)c(1)	16	5495.53	4.36	0.04	6470.99

**Table 3:** Parameter estimates (95 % CI) obtained from the selected models of abundance of Control populations in 2013/2014 and 2014/2015 from Table 2. S: 1‐month survival probability, p: probability of initial capture during a trapping session, c: probability of recapture during a trapping session, conditional on initial capture

Component	Level	Mean (95% CI)
Winter 2013/2014		
S(Elevation)	Survival high elevation	0.81 (0.77, 0.84)
	Survival low elevation	0.69 (0.66, 0.73)
p(Elevation)	Trappability high elevation	0.19 (0.16, 0.23)
	Trappability low elevation	0.27 (0.39, 0.45)
c(Elevation)	Recapture high elevation	0.42 (0.39, 0.45)
	Recapture low elevation	0.46 (0.43, 0.48)
Winter 2014/2015		
S(Elevation)	Survival high elevation	0.56 (0.50, 0.62)
	Survival low elevation	0.47 (0.41, 0.52)
p(1)	Trappability	0.30 (0.27, 0.34)
c(1)	Recapture elevation	0.46 (0.44, 0.48)

**Figure 5:**
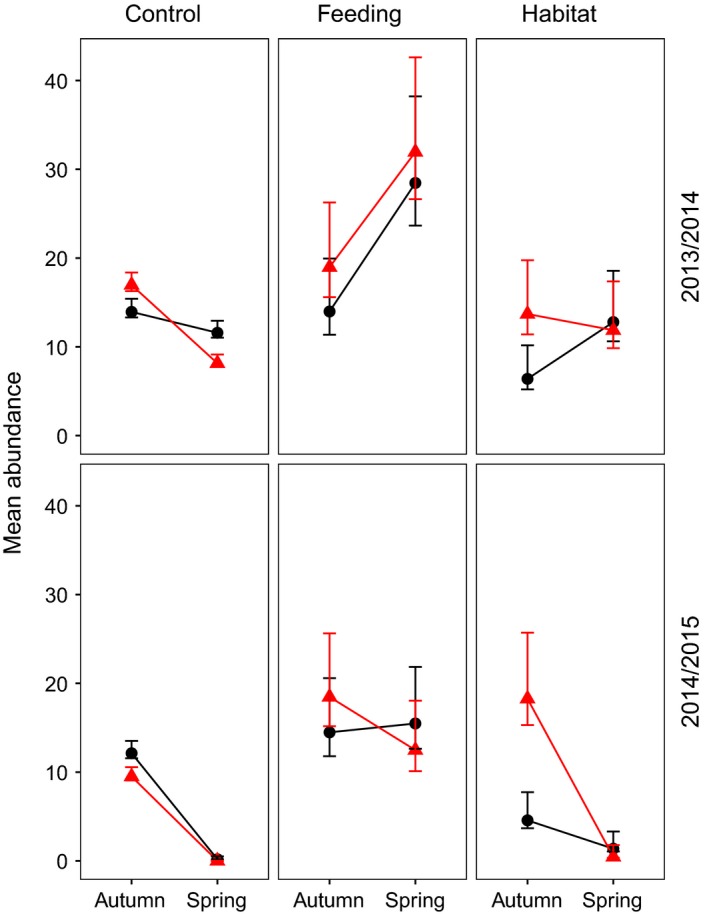
Mean abundance of bank voles with 95 % CI per plot (3600 m^2^) in autumn and spring for all Treatment populations (Control, Feeding and Habitat) during the winters 2013/2014 and 2014/2015. The high elevation populations are presented with black circles and lines, and low elevation populations with red triangles and lines. Estimates were obtained from the robust design model highlighted in Table 4

For the manipulated plots, we only had trapping sessions in autumn and spring. Feeding and Habitat populations started autumn with the same abundance level in both winters. The Feeding populations increased 83 % over the 2013/2014 winter. In contrast, their populations declined markedly over the 2014/2015 winter, with only 15 % remaining by May (Figure 5). The Habitat populations increased 23 % over the 2013/2014 winter, but crashed over the 2014/2015 winter (with voles being captured on only two of six plots in May 2015). The crash on the Control plots was even more dramatic, with voles being captured on only 1 of 14 plots. In contrast, voles were captured on all six Feeding plots (Appendix). The populations that went extinct over the 2014/2015 winter remained so throughout the summer of 2015 (K. Johnsen, unpublished material).

### 3.3 Survival

**Figure 6:**
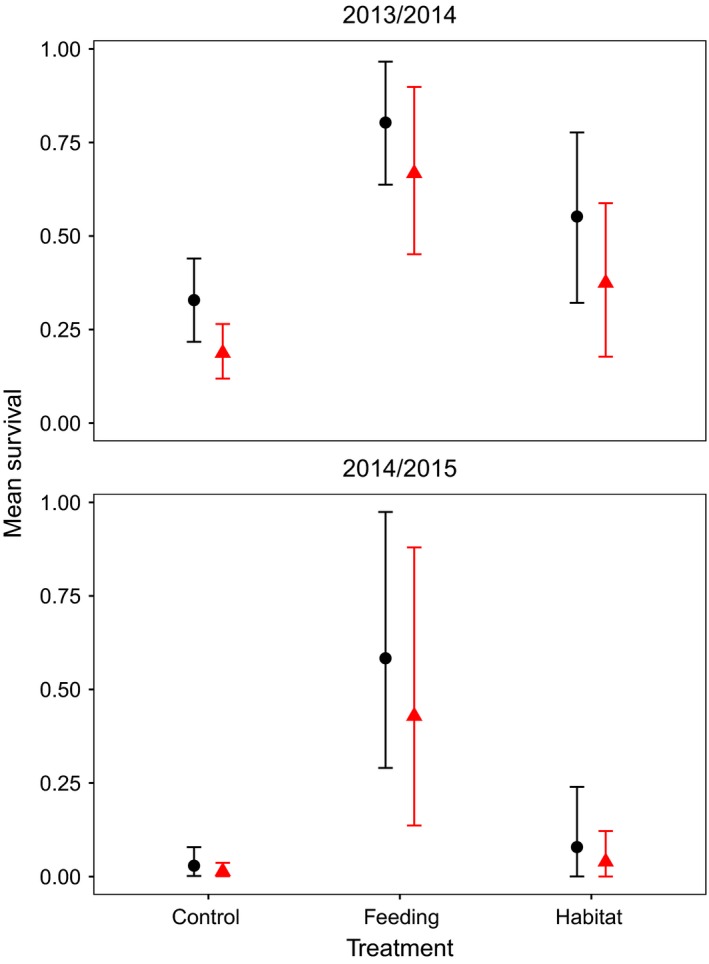
Mean 6‐month, autumn to spring survival (95 % highest posterior density intervals) of bank voles for all Treatment populations (Control, Feeding and Habitat) during winters of 2013/2014 and 2014/2015. The high elevation populations are presented with black circles and lines, and the low elevation populations with red triangles and lines. Estimates were obtained from the MCMC estimation of the best model highlighted in Table 3

The most parsimonious model selected for 6‐month survival (i.e., from November/December to May) included an interaction effect of Time and Treatment, in addition to an Elevation effect (Table 4). Over both winters, survival was ~4.5 times higher on the Feeding populations than on the Control populations at both Elevations (Figure 6). Over the 2014/2015 winter, survival at low elevation was ~30 times higher on the Feeding than on the Control populations, and at high elevation survival was ~20 times higher on the Feeding than on the Control populations (Figure 6). Over the 2014/2015 winter, the survival on the Feeding was also higher than that on the Habitat populations, at both elevations. Across all treatments and years, survival was ~1.4 times higher at the high than the low elevation populations.

**Table 4:** Model selection for the modeling of winter survival and abundance of bank voles on Treatment populations (Control, Feeding and Habitat) by use of the robust design model with Huggins parameterization. In this analysis, capture–recapture data was available only in autumn and spring (autumn 2013, spring and autumn 2014, and spring 2015). This model included both winters (2013/2014 and 2014/2015) together, using a 6‐month interval between primary sessions. Only the five best AIC models are shown, and the model used for MCMC estimation is highlighted. The model structure for GammaPrime (γ'), GammaDoublePrime (γ”), capture probability (c) and recapture probability (p) are not shown, because it was the same across all models. Elevation is the elevation level (low, high), Treatment is the experimental treatment (Control, Feeding, or Habitat); Time corresponds to the primary trapping sessions every 6 months

Model	npar	AICc	ΔAICc	AICc weight	Deviance
S(Elevation + Time*Treatment)	**19**	**7629.35**	**0.00**	**0.97**	**6463.05**
S(Time*Elevation*Treatment)	27	7637.78	8.44	0.01	6455.12
S(Time*Treatment)	18	7637.92	8.57	0.01	6473.66
S(Time + Treatment*Elevation)	17	7648.83	19.48	0.00	6486.61
S(Treatment + Time*Elevation)	17	7650.16	20.81	0.00	6487.93

## 4 Discussion

Our objective was to understand the role of three key extrinsic factors (environmental stability, food, and habitat structure) as explanatory factors explaining or contributing to winter crashes in the bank vole. The crash occurring over the winter of 2014/2015 was gradual, not instantaneous. The populations on 13 of 14 Control plots, and four of six Habitat plots went extinct during this winter, whereas some voles on all of the Feeding plots survived. Hence, food, and not habitat structure or environmental stability, seems to have prevented a crash.

The environments of the plots at high and low elevations indeed showed the expected differences, with those at high elevations having greater stability: lower temperatures, deeper snow, and longer lasting snow cover than those at low elevations. The reduced environmental stability at low elevations relative to high elevations was particularly pronounced in the 2013/2014 winter, when the low elevation sites experienced a mild mid‐winter warming that did not occur at high elevations. Hence, the higher survival we observed at high elevation might be explained by improved subnivean conditions. Ims, Yoccoz, and Killengreen (2011) found that the amplitude of the Norwegian lemming *Lemmus lemmus* outbreaks increased with elevation, possibly owing to improved subnivean winter habitat conditions, leading to reproduction under the snow. Even though the lemming cycles tend to have a sharper, more saw‐toothed, pattern than vole cycles (Turchin, Oksanen, Ekerholm, Oksanen, & Henttonen, 2000), and in spite of the fact that lemmings occurring predominantly in harsh tundra and alpine habitats, we might expect that similar mechanisms related to elevation will affect lemmings and vole populations similarly. However, even though winter conditions may cause some of the variation in vole survival, we did not find that adverse winter conditions caused the vole population to crash in 2014/2015. The within‐year variation in winter conditions between elevations was higher than the between‐year variation. Nevertheless, no Control plots at either high or low elevation went extinct the first year, whereas almost all went extinct the second year.

We were not able to increase survival by improving the winter habitat structure with straw. Korslund and Steen (2006) improved habitat structure (i.e., limiting icing in subnivean space) by adding aluminum sheets prior to snowfall and found that they increased overwinter survival in the tundra vole *Microtus oeconomus*. However, a complicating factor with their study was that the voles they had introduced to their experimental sites were not “natural crash phase” voles. Similar habitat benefits have been suggested in other studies on the winter ecology of voles (Aars & Ims, 2002; Ims et al., 2011; Kausrud et al., 2008). In contrast, Hoset, Le Galliard, and Gundersen (2009) found that the amount of ice accumulation did not affect winter survival of enclosed populations of tundra voles as they simply avoided ground ice by moving their home range, thus increasing home range overlap and reducing the negative effect of unstable winter weather through social behavior. However, if the entire landscape is affected by subnivean icing, there will be nowhere to move. In our study, the population decline and crash during the second winter was gradual rather than sudden as expected if the crash was caused by freeze‐thaw events and icing. The lack of any Habitat effect can thus be due to lack of freeze‐thaw events during the study winters. However, even in the absence of such freeze‐thaw events, the population crashed in 2014/2015. Our habitat structure manipulation was thus not strong enough to prevent a naturally occurring vole population crash.

Our results confirmed that supplemental feeding during winter had a positive effect on the population abundance and winter survival. Vole abundance increased from autumn to spring with supplementary feeding the first winter (2013/2014), indicating that immigration had to have occurred as we observed no reproduction. Immigration has been shown to increase with supplemental feeding (Gilbert & Krebs, 1981; Prevedello, Dickman, Vieira, & Vieira, 2013; Schweiger & Boutin, 1995). However, our survival estimates are based on the recapture of known individuals and thus do not include immigrants.

The effect of the feeding treatment on survival may have been mediated directly by higher food quality and/or quantity or indirectly through interactions with other factors such as disease or predation. First, the quantity (i.e., unlimited supply) and quality of food could have a direct effect on the winter survival of voles (Cole & Batzli, 1978). Schweiger and Boutin (1995) found an effect of supplementary feeding of sunflower seeds on the survival of red‐backed voles *Myodes rutilus* over winter and Rémy (2011) found positive effects of food on population growth mediated through changes in social behavior in bank voles. Boonstra and Krebs (2006) found no effect of food addition on red‐backed voles in the Yukon, probably due to inappropriate food (rabbit pellets) for voles. Based on the results of Schweiger and Boutin (1995), they concluded that the interaction between winter conditions and food was important for red‐backed vole population dynamics. We used mostly oats but also 20 % sunflower seeds and assumed that any supplement would be an improvement on the food naturally available in winter.

The effect of food supplementation could be mediated through interactions with other factors not controlled in our experiment. Predation could be an important factor as predator populations were expected to be high after two years of high vole densities (Korpimäki et al., 1991). For instance, Huitu et al. (2003) showed that winter food supplementation increased survival of field voles *Microtus agrestis*, but only in the absence of predators (see also Prevedello et al., 2013). As all our populations were subject to predation and the location of Control and manipulated populations were intermixed, there is no reason to expect that predators would not also find the Feeding populations. Therefore, food supplementation must have increased survival in spite of the presence of predators. Food supplementation may also have altered vole behavior. Supplemental feeding usually results in smaller home ranges (Boutin, 1990), and increased movement has been shown to increase predation (Andreassen & Ims, 2001; Andreassen, Stenseth, & Ims, 2002; Ims & Andreassen, 2000; Norrdahl & Korpimäki, 1998). With unlimited access to food in fixed locations, the voles did not have to move around as much to forage and thus were possibly less exposed to predators. Bank voles are known to hoard food (Mappes, 1998). If hoarding was done at locations that were unavailable to mustelids and other predators, it could reduce vole exposure to predators even more.

In addition to predation, the effect of food supplementation may also be mediated by diseases. Higher vole abundance may imply higher stress levels and higher susceptibility and exposure to parasites and disease outbreaks. If individuals are infected with diseases, survival during winter could be more difficult owing to low immunity under harsh conditions. For example, Kallio et al. (2007) found that the winter survival of bank voles was 4.5 times lower when the animals were infected with hantavirus. Moreover, individuals congregate more when they are supplemented with food, leading to a possible increase in pathogen transmission (Becker, Streicker, & Altizer, 2015; Forbes et al., 2015). However, supplementary feeding may also result in animals in better physical condition, with an improved immune system resulting in higher chances of surviving the winter even if they are infected (Ostfeld, 2008). Pedersen and Greives (2008) managed to prevent a population crash in *Peromyscus* by combining supplementary feeding with the removal of intestinal nematodes with drugs, whereas single‐factor manipulations only reduced the population crash.

## 5 Conclusion

Winter conditions may be important for vole survival during winter and may explain the recent disappearance of voles following peak years. However, our results suggest that factors other than winter conditions mediate winter crashes in cyclic vole populations. We show that food availability is crucial for winter survival of bank voles, which supports The Food Limitation Hypothesis. Whether food availability affects winter survival of bank voles in a direct density dependent manner via starvation, or indirectly through decreased susceptibility to predation or diseases, remains to be investigated. Future studies on winter ecology of voles should focus on how food affects the behavior of voles during winter, and how it interacts with other factors such as predation and disease.

## Conflict of Interest

None declared.

## Appendix 1 Surviving winter: Food, but not habitat structure, prevents crashes in cyclic vole populations

### 1.1

Kaja Johnsen, Rudy Boonstra, Stan Boutin, Olivier Devineau, Charles J. Krebs, Harry P. Andreassen

Number of voles per trap in each plot estimated by minimum number of voles known to be alive (MNA)/number of traps for each primary trapping session. Most plots included 16 traps and covered 3,600 m^2^. An individual was defined as present in a plot at a primary session *t* if it was caught at least once in the plot at a secondary trapping session. Furthermore, an individual was also assumed to be alive and present in the plot at a primary trapping session *t*_0_ if it had been caught at least once before in a primary session *t*_0−*x*_ and once in a later primary trapping session *t*_0*+x*_. Primary sessions from October and June both years were used to estimate MNA for November and May.

The figure shows that almost all plots in Control and Habitat went extinct the winter 2014/2015, that is: We captured no voles during the spring trapping. The High elevation populations are presented with black circles and lines, and Low elevation populations with red triangles and lines.
